# Biosynthesis of Apigenin Glucosides in Engineered *Corynebacterium glutamicum*

**DOI:** 10.4014/jmb.2401.01017

**Published:** 2024-03-14

**Authors:** Obed Jackson Amoah, Samir Bahadur Thapa, Su Yeong Ma, Hue Thi Nguyen, Morshed Md Zakaria, Jae Kyung Sohng

**Affiliations:** 1 Department of Life Science and Biochemical Engineering, Sun Moon University, Asan-si 31460, Republic of Korea; 2 Department of Pharmaceutical Engineering and Biotechnology, Sun Moon University, Asan-si 31460, Republic of Korea

**Keywords:** *Corynebacterium*, glucosylation, apigenin, glycosyltransferase

## Abstract

Glucosylation is a well-known approach to improve the solubility, pharmacological, and biological properties of flavonoids, making flavonoid glucosides a target for large-scale biosynthesis. However, the low yield of products coupled with the requirement of expensive UDP-sugars limits the application of enzymatic systems for large-scale. *C. glutamicum* is a Gram-positive and generally regarded as safe (GRAS) bacteria frequently employed for the large-scale production of amino acids and bio-fuels. Due to the versatility of its cell factory system and its non-endotoxin producing properties, it has become an attractive system for the industrial-scale biosynthesis of alternate products. Here, we explored the cell factory of *C. glutamicum* for efficient glucosylation of flavonoids using apigenin as a model flavonoid, with the heterologous expression of a promiscuous glycosyltransferase, YdhE from *Bacillus licheniformis* and the endogenous overexpression of *C. glutamicum* genes *galU1* encoding UDP-glucose pyrophosphorylase and *pgm* encoding phosphoglucomutase involved in the synthesis of UDP-glucose to create a *C. glutamicum* cell factory system capable of efficiently glucosylation apigenin with a high yield of glucosides production. Consequently, the production of various apigenin glucosides was controlled under different temperatures yielding almost 4.2 mM of APG1(apigenin-4'-O-β-glucoside) at 25°C, and 0.6 mM of APG2 (apigenin-7-O-β-glucoside), 1.7 mM of APG3 (apigenin-4',7-O-β-diglucoside) and 2.1 mM of APG4 (apigenin-4',5-O-β-diglucoside) after 40 h of incubation with the supplementation of 5 mM of apigenin and 37°C. The cost-effective developed system could be used to modify a wide range of plant secondary metabolites with increased pharmacokinetic activities on a large scale without the use of expensive UDP-sugars.

## Introduction

Flavonoids are naturally occurring secondary metabolites from plants and fungi generally characterized by a 15-carbon skeleton with two phenyl rings (A and B) that are linked together by a heterocyclic ring [1, 2]. Additionally, flavonoids are severally subcategorized based on their chemical structure, which includes, flavones, chalcones, isoflavones, anthocyanins, flavonols, and flavanones [3]. The flavone subclass of flavonoids consists of a backbone of 2-phenylchromagen-4-one with a double bond between the C2 and C3 [4]. Common flavones like apigenin constitute an integral part of the human diet, displaying anticancer, anti-inflammatory, hypolipidemic activity, heart disease prevention, and antitumor benefits [4-9]. Furthermore, apigenin has been determined to show additional, anti-aging, anti-fungal, anti-tumor, anti-proliferative, and anti-oxidant activity against reactive oxidative species (ROS), thus, making apigenin highly valuable compounds for food and pharmaceutical industries [10-12].

However, the poor solubility, bioavailability, and stability of apigenin limit, its applications as potent functional materials in the food, cosmetic, and pharmaceutical industries despite their potential abilities [13-15]. Therefore, the need to modify apigenin has been of interest in recent years. Among the post-modification applications, glucosylation, which is the functional modification of natural flavonoids by the introduction of sugar moieties to the flavonoid’s core, has broadly been known to increase the bioactivity, conformation, solubility, half-life, and pharmacokinetic properties [16-18]. For instance, apigenin 7-O-β-glucoside as synthesized by Thuan *et al*. 2018, appeared to have several additional properties, including higher antioxidant, solubility, and anti-proliferative activities [19]. Additionally, owing to their prospective pharmacological and food applications, flavonoids, including apigenin have become a target for large-scale synthesis [20]. There have been successful attempts to synthesize various flavonoid glucosides by the use of enzymatic reactions by either glycosyltransferase (GT) or chemical synthesis. Whereas GTs provide a flexible forum and wide range of substrates, current research utilizes an in vitro enzymatic system, which requires the supply of expensive activated sugar donors such as UDP-glucose, making it not feasible for industrial and large-scale synthesis [21]. Similarly, the destruction of substrates, low yield, formation of undesirable anomers, and its laboriousness have led to the preparation of flavonoid glucosides by chemical synthesis nearly impossible [12]. Therefore, to overcome the challenges that hinder the large-scale preparations of flavonoid glucosides, the engineering of microorganisms such as *Escherichia coli*, *Saccharomyces cerevisiae*, and *C. glutamicum* has been employed for the biosynthesis of these natural products [22, 23]. *C. glutamicum* in particular, possesses a cell factory that is enhanced for the industrial-scale synthesis of products, hence it has been referred to as the workhorse of the biotechnology industry [24]. Thus making it a better platform for the cost-effective synthesis of flavonoid glucosides on an industrial scale.

The Gram-positive, facultatively anaerobic, non-sporulating soil bacteria, *C. glutamicum*, has been a major producer of amino acids such as L-glutamate and L-leucine ever since its discovery in the late 1950s in Japan [25, 26]. Due to its status as GRAS (generally regarded as safe) bacteria, *C. glutamicum* has been preferred for the large-scale fermentation of valuable compounds such as hyaluronic acid, glutathione, and amino acids for the pharmaceutical, cosmetic, and food industries [27, 28]. Additionally, the product spectrum of *C. glutamicum* has greatly increased to include biofuels such as ethanol, carotenoids, muconic acid, and isobutanol [25, 29-33]. On top of the numerous advantages, *C. glutamicum* has been successful in white biotechnology due to its ease of cultivation, high substrate susceptibility, its ability to use different sugars as carbon sources, and the availability of strategies for metabolic engineering [34, 35]. The cell factory of *C. glutamicum* has been explored for the production of glucosylated carotenoids C_50_ and C_40_, cadaverine, anthocyanins, and other pharmaceutical-relevant flavonoids [36, 37].

In the present study, a simple strategy for the engineering of *C. glutamicum* by the overexpression of the homologous UDP-D-glucose pathway genes, phosphoglucomutase (*pgm*) (Gene ID; CP025533.1) and UDP-D-glucose pyrophosphorylase (*galU1*) (Gene ID; HE802067.1), to enhance the activated sugar pool and the subsequent heterologous expression of *ydhE* (Gene ID; AAU3977.1), a promiscuous enzyme from *Bacillus licheniformis* was employed. We used the constructed strain to probe apigenin for efficient biosynthesis of flavonoid glucosides. Furthermore, the effect of overexpression of homologous genes involved UDP-D-glucose biosynthesis pathway was studied for efficient glucosylation and maximum yield of glucosides. The engineered strain of *C. glutamicum* was able to produce the highest titers of apigenin glucosides reported up to date.

## Materials and Methods

### Chemicals and Reagents

Apigenin was of 98% purity and purchased from Takara Chemical Industry (TCI, Japan). Restriction enzymes, *taq* polymerase, pGEM-T Easy cloning vector, and related reagents were purchased from either Takara Bio Inc.,(Japan) or Promega (USA). All other chemicals and reagents such as HPLC-grade acetonitrile and deionized water were of high-grade and commercial sources.

### Bacterial Strains and Culture Conditions

The list of all bacteria strains and plasmids are listed in the supplementary file; Table S1. *Escherichia coli* (MRF)(*E. coli*) (Stratagene, USA) was employed for all gene clone cloning and laboratory procedures. Luria-Broth (LB), supplemented with either 50 μg/ml of kanamycin or ampicillin (when appropriate) was utilized for the culture and growth of all *E. coli*. When necessary, 10 g/l of agar (Sigma, USA) was added to prepare the LB-agar media plate. *Corynebacterium glutamicum* ATCC 13032 (*C. glutamicum*) was used as the expression host for the biosynthesis of flavone glucosides. *C. glutamicum* was routinely cultured in Brain Heart Infusion (BHI) medium (10 g/l tryptone, 5 g/l yeast extract, 10 g/l brain heart infusion Broth, and 10 g/l NaCl) (Difco, USA) at 30°C with shaking at 200 rpm. To obtain transformants, BHIS (BHI medium supplemented with 91 g/l sorbitol) medium with appropriate antibiotics was used.

### Genetic Modification and Plasmid Construction of Recombinant *C. glutamicum*

The plasmid pSKSM-YdhE (pSKSM-Y [38]), which harbors the YdhE, was confirmed by the traditional restriction digestion at the *Bam*HI and *Xba*I. To construct plasmid pSKSM-YdhE-GalU1-Pgm (pSKSM-YGP), the *galU1* and *pgm* encoding UDP-glucose pyrophosphorylase and phosphoglucomutase respectively were PCR amplified using *C. glutamicum* genomic DNA, extracted with Promega DNA extraction kit (Madison, Promega, USA) and using primer pairs galU1-fwd/rev, pgm-fwd/rev respectively (Table S2). The generated PCR products were verified and separated with electrophoresis in agarose gel (0.6%), purified, and ligated into the linearized pSKSM-Y (for cloning of *galU1*) and pSKSM-YG (for cloning of *pgm*) at the XbaI/NotI and NotI/PstI restriction sites respectively.

Preparation of electrocompetent cells and transformation of recombinant plasmids into *C. glutamicum* was done as previously described [39]. In brief, a single colony of fresh *C. glutamicum* was inoculated in 5 ml of BHIS media for 12 h. After then, 500 μl of seed culture was inoculated in 50 ml of fresh BHIS media with the supplementation of 1 ml/l Tween 80 with shaking at 200 rpm. When OD_600_ reached 0.8, cells were chilled on ice, centrifuged, and washed four times with 10% glycerol. 150 μl of cell suspension in 1 ml of 10% glycerol were stored at -80°C to be used for transformation. For transformation, an electroporator (Bio-Rad) at 25 μF, 200 W, and 2.5 kV, yielding a pulse duration of ~5 ms was used. After electroporation, cells were mixed with 1 ml pre-warmed BHIS in the cuvette, transferred to a 2-ml microcentrifuge tube, and heat-shocked at 46°C for 6 min in a water bath. Cells were then transferred to a 15 ml culture tube (VWR), incubated for 90 min at 30°C, and plated on BHIS plates containing kanamycin (50 mg/l). Positive colonies were verified by restriction enzyme digestion and gene sequencing. (Genotech, Republic of Korea).

### Fermentation Method and Conditions

Recombinant *C. glutamicum* strains of harboring plasmid pSKSM-Y and pSKSM-YGP were used for the biotransformation of flavones. Seed cultures of recombinant *C. glutamicum* were prepared in 6 ml BHI medium with the supplementation of 50 μg/ml of kanamycin and incubated for 12 h at 30°C with 200 rpm. Then after, 500 μl of seed culture was inoculated in 50 ml of BHIS media with 4 g/l of either glucose or sucrose supplied when appropriate. Optical density was monitored until OD_600_ reached 0.6-0.8. Isopropyl-β-*D*-1-thiogalactopyranoside (IPTG) was then added to the final concentration of 0.5 mM for the induction of heterologous pathway protein expression. Cells were then incubated until OD_600_ reached 2.0. Since apigenin is insoluble in water, all substrates were dissolved in dimethyl sulfoxide (DMSO) to a final concentration of 100 mM stock, which was then exogenously supplied, with varying concentrations and time intervals for the optimization of the biotransformation of the glucosides.

### Extraction, Purification, and Analytical Procedure

Products were extracted with single volumes of ethanol and ethyl acetate (*v/v*= 1;1:1). After vigorous stirring, it was concentrated with a rotary evaporator. Products were subjected to high-performance liquid chromatography–photodiode array (HPLC-PDA) analysis with detection at 339 nm. Acetonitrile (ACN) and water (0.1%trifluoroacetic acid) were used as the mobile phase. ACN concentrations were as follows: 20%, (0–5 min); 50%, (5–10 min); 70%, (10–15 min); 90%, (15–20) min; and 10%, (20–25 min); with a flow rate of 1 ml/min.

To purify compounds, 3,000 UPLC (Thermo Fisher Scientific, USA) with a C18 column (YMC-Pack ODS-AQ (250 mm × 20 mm I.D., 10 μm, Japan) connected to a UV detector at 273 nm using a 40 min binary program with 100% triple-distilled H_2_O and ACN (10%, (0–5 min); 40%, (5–15 min); 90%, (15–30 min); and 10%, (30–40 min); at a flow rate of 10 ml/min was used.

Purified samples were dissolved in D_2_O, lyophilized, and completely dried. They were then dissolved in 600 μl dimethyl sulfoxide-*d6* (DMSO-*d6*) for nuclear magnetic resonance (NMR) analysis. Extracted products, together with their standards were subjected to NMR analysis. Compounds were characterized by 900 MHz Avance II 900 Bruker BioSpin NMR spectrometry (Bruker, USA) using a Cryogenic TCi probe (5 mm). All metabolites were analyzed with Topspin 3.1 software (Bruker), and further processed using MestReNova 12.0 software (Mestrelab Research S.L., Spain).

## Results

### Effect of Increased UDP-Glucose Pool on in vivo Bioconversion

Intracellular NDP-sugar is used as a donor by the glycosyltransferase enzyme to the aglycon acceptor in the in vivo system. UDP-glucose serves as a co-substrate and its intracellular amount could have a critical impact on the biosynthesis of flavone glucosides. Previous studies have demonstrated the inefficiency of heterologous expression of the UDP-glucose pathway genes in *C. glutamicum* due to incoordination with the native UDP-glucose biosynthesis network causing translational imbalance, however, the overexpression of endogenous UDP-glucose pathway genes greatly increases UDP-glucose levels for cyaniding glucosylation [40, 41]

**Fig. 1:**
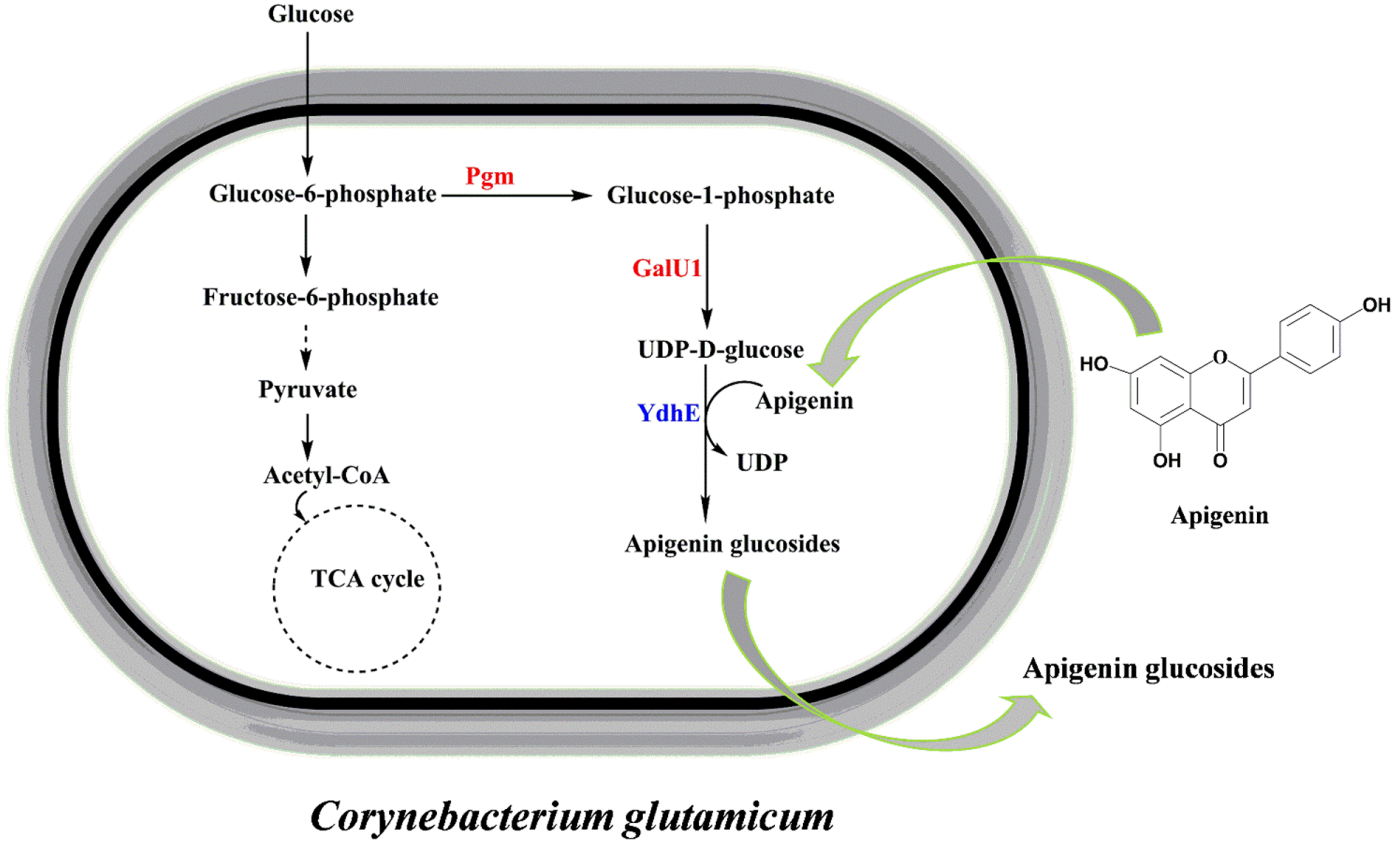
A general schematic pathway representation for the production of apigenin glucoside derivatives in engineered *C. glutamicum* with the supplementation extracellular supplementation of glucose for largescale biosynthesis by fermentation.

**Fig. 2:**
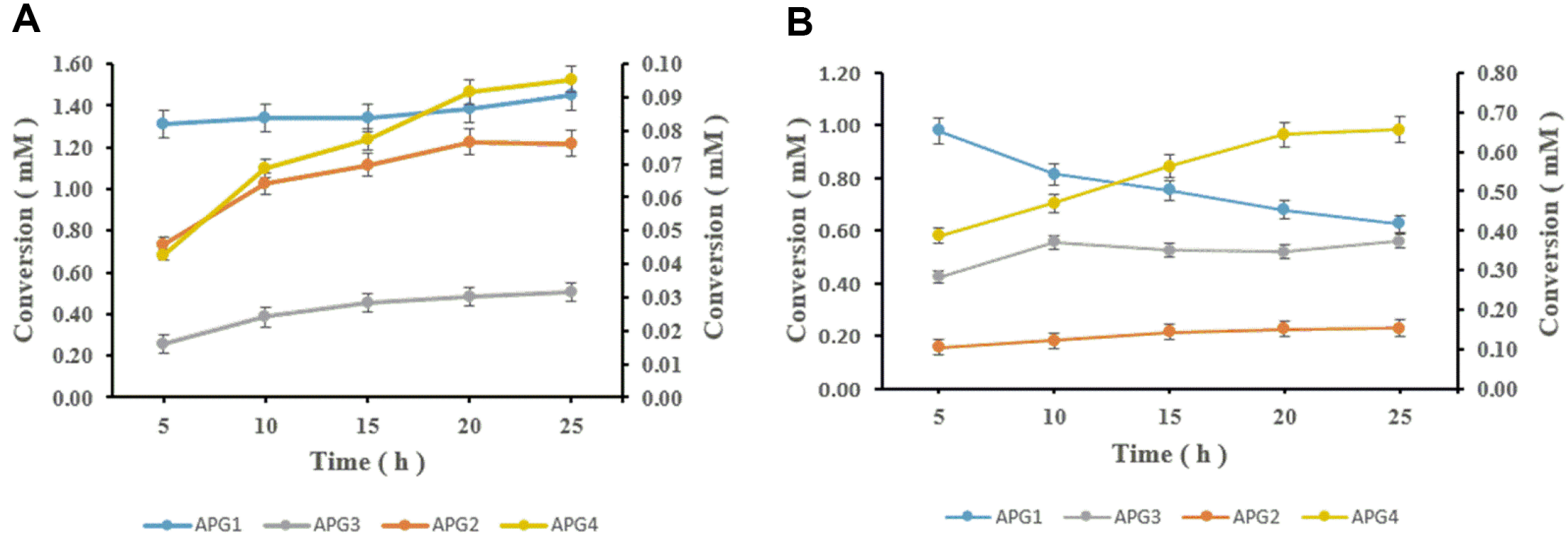
Production pattern of glucosylated derivatives APG1 (apigenin-4'-β-O-glucoside), APG2 (apigenin-7-β-O-glucoside), APG3 (apigenin-4'-7-β-O-diglucoside) and APG4 (apigenin 4'-5-β-O-diglucoside )with strain (A) CgIBR-1 and (B) CgIBR-2. In the absence of substrate and high amount of UDP-glucose produced, YdhE uses APG1 as a substrate for the production of APG2, APG3, and APG4. Hence the decrease in APG1 and the observed increase in APG2 and APG3 and APG4. The primary axis represents the conversion of APG1 and APG3 whilst the secondary axis represents the conversion of APG2 and APG.

We aimed to synthesize apigenin glucosides using native UDP-glucose in *C. glutamicum* as a sugar donor by YdhE (Fig. 1). Cell cultures of *C. glutamicum* harboring pSKSM-Y and pSKSM-YPG were prepared for biotransformation as mentioned in materials and methods. Bioconversion was monitored in a five-hour interval to ascertain the production efficiency of the strains toward the maximum glucosylation of all possible apigenin glucoside derivatives. The results showed a varying pattern of product accumulation between the two strains after 25 h of incubation. Whereas CgIBR-1, *C. glutamicum* harboring expressed YhdE (pSKSM-Y) showed a continuous increase in all products, APG1 from 1.3 mM to 1.41 mM, APG2 from 0.04 mM to 0.07 mM, APG3 from 0.26 mM to 0.5 mM, and APG4 from 0.04 mM to 0.09 mM, CgIBR-2, *C. glutamicum* harboring expressed YhdE and homologous *pgm* and *galU1* (pSKSM-YPG) showed a rapid decrease in APG1 from 0.9 mM to 0.6 mM. Surprisingly, the formation of APG2, APG3, and APG4 drastically increased as follows; APG2 from 0.10 mM to 0.15 mM, APG3 from 0.42 mM to 0.56 mM, and APG4 from 0.38 mM to 0.65 mM (Fig. 2). The pattern of glucosylation of these glucosides suggests the formation of APG1 is favored by low concentration of UDP-glucose whereas high concentration of UDP-glucose favors the formation of APG2, APG3 and APG4. Similarly, the increased concentration of UDP-glucose pool in CgIBR-2 in the presence of a low concentration of substrate enables YdhE to use APG1 for the synthesis of the other products, hence the observed pattern.

### Effect of Increased Substrate Concentration on Apigenin Glucosides Production

The biotransformations of *C. glutamicum* were optimized after the confirmation of their ability to convert apigenin to its glucoside derivatives. Moreover, the decline of the conversion of APGI in CgIBR-2 was thought to be the abundance of UDP-D-glucose in the presence of low substrate concentration enabled YdhE to use APG1 as a substrate to produce APG2, APG3, and APG4.

**Fig. 3:**
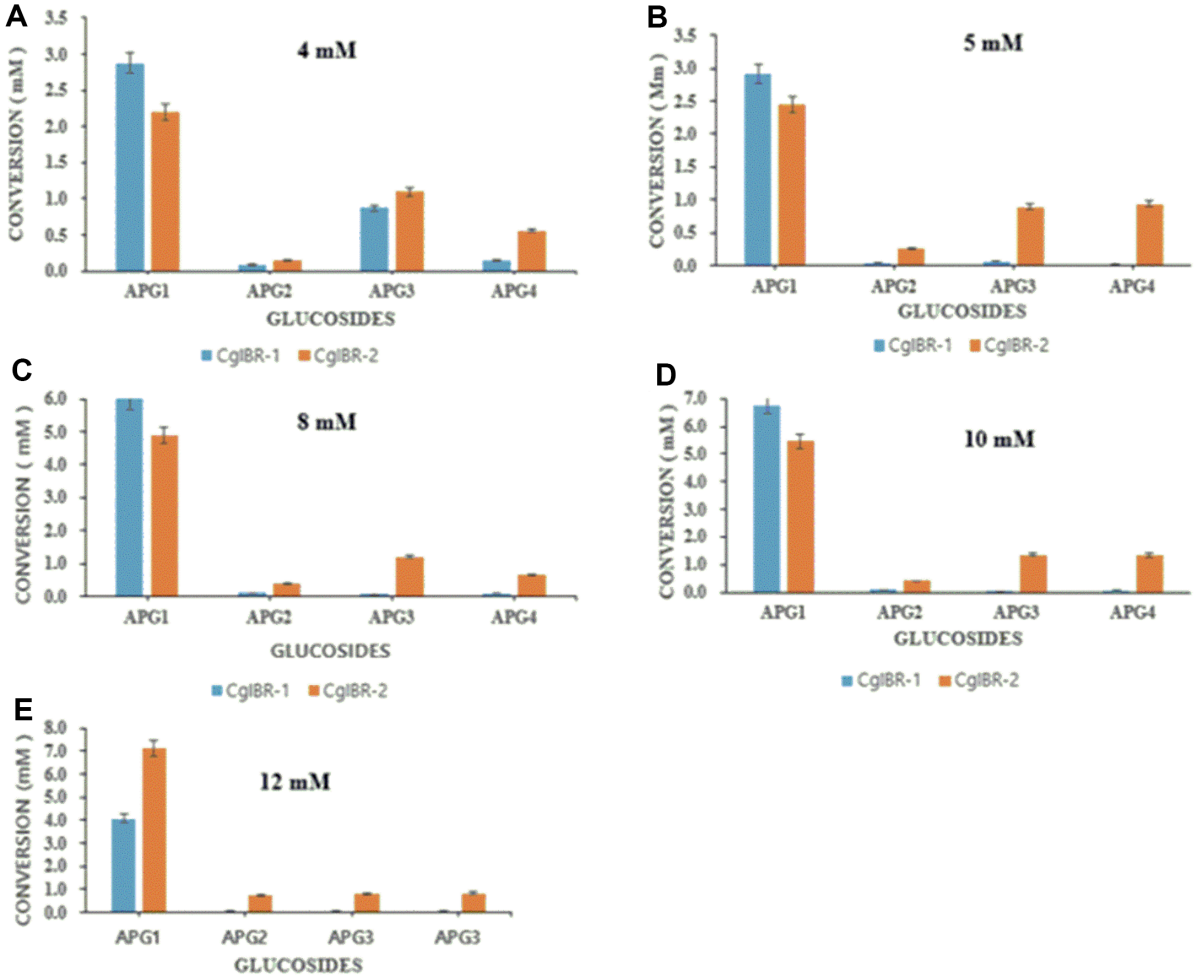
Production profiles of apigenin-*O*-glucosides with the supplementation of different concentrations of apigenin with the two *C. glutamicum* engineered strains CgIBR-1 and CgIBR-2 after 25 h of incubation.

Next, we set out to determine the optimum substrate susceptibility of the two developed strains. A set of apigenin concentrations 4, 5, 8, 10, and 12 mM was exogenously supplemented to the culture medium and monitored until 25 h. As shown in Fig. 3, CgIBR-1 produced the highest concentration of APG1. APG1 proved to be the major product in both strains. However, the bioconversion of APG2, APG3, and APG4 in strain CgIBR-1 decreased with increased concentration of apigenin and remained <5 % when more than 5 mM was supplemented to the culture medium. Consequently, in the case of APG1, the strain CgIBR-2 showed a higher conversion with increased concentration. Even though CgIBR-2 produced a higher amount of ~7 mM of APG1 with the supplementation of 12 mM apigenin (Fig. 3E), the concentration of APG2, APG3, and APG4 did not significantly change. The highest production was APG1, APG2, APG3, and APG4 of 2.5 mM, 0.2 mM, 1.2 mM, and 0.9 mM respectively, was observed when 4 mM of apigenin was supplemented to the culture medium. Additionally, minimal apigenin residues were observed in cultures with 5 mM compared to the cultures with higher concentrations of apigenin (Figs. 3B and S2).

### High-Level Selective Production of Apigenin Glucosides with Varying Temperatures

**Fig. 4:**
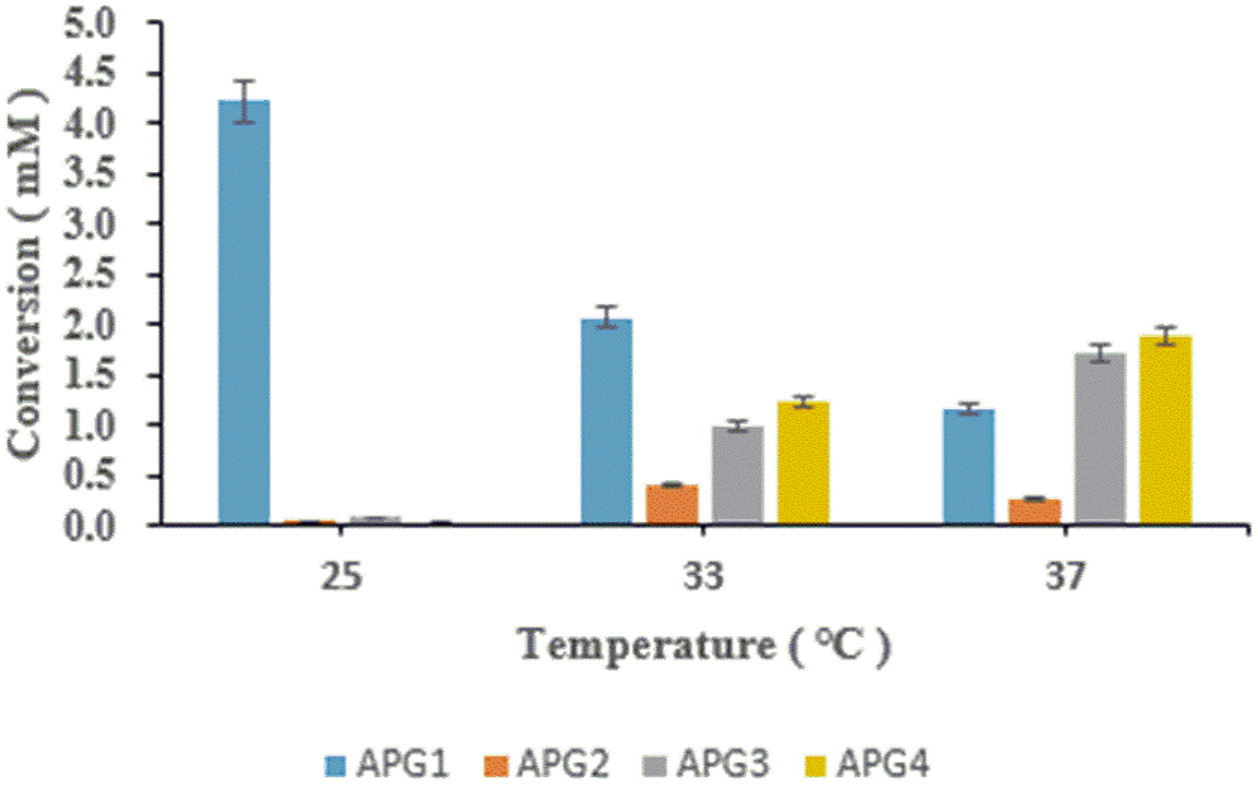
Temperature control for the selective production of apigenin glucosides with CgIBR-2. Lower temperatures favor the production of APG1 whilst higher temperatures favor the production of diglucosides.

**Fig. 5:**
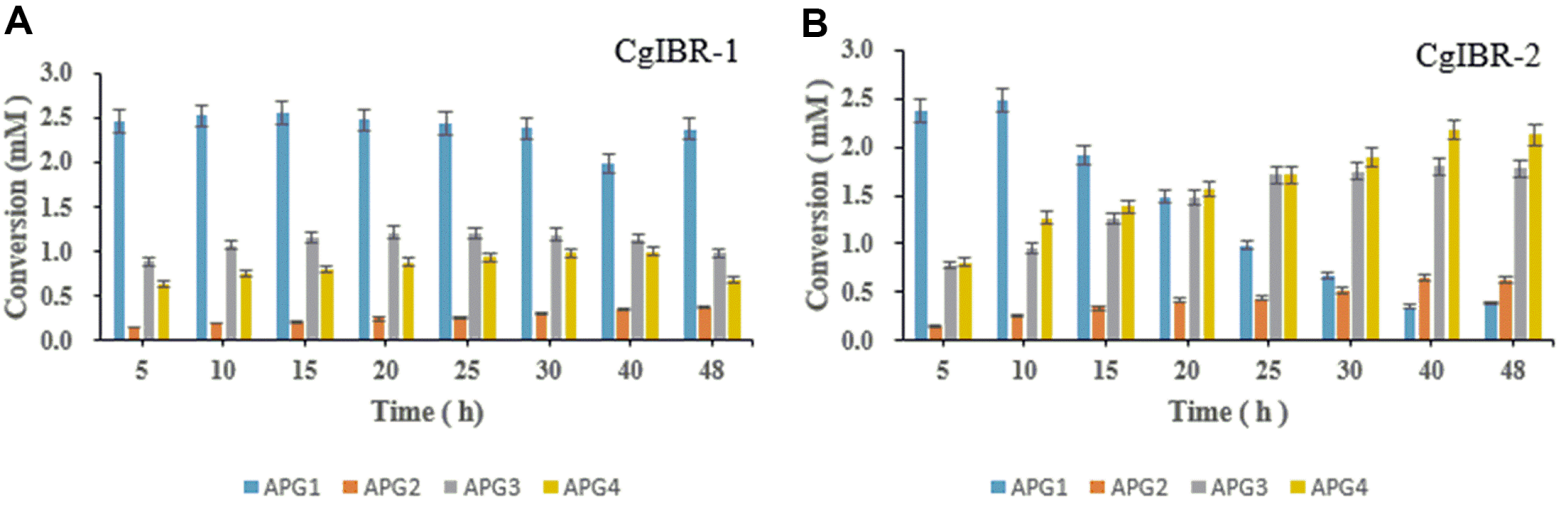
Comparative production profiles at 37°C of (A) Strain Cg-IBR compared to (B) CgIBR-2 after 48 h of incubation. Error bars show the mean deviation of three distinct experiments.

The production profiles of various fermentation products in *C. glutamicum* were recently revealed to be independent of growth temperature and fermentation at different temperatures could maximize the efficiency of its cell factory towards different target products [42]. To control and elevate the production of different glucosides in strain CgIBR-2, cells were cultured and induced as mentioned in the materials and methods section. The comparative conversion rates analyzed by HPLC revealed a varying impact on the formation of products with different fermentation temperatures in *C. glutamicum*. At 25°C, maximum production of APG1 of ~4.2 mM was achieved. Furthermore, the concentration of APG1 was significantly reduced at 33°C and maximum yields of the diglucosides APG3 and APG4 were achieved when cells were fermented at 37°C yielding ~1.8 mM and 2.0 mM respectively (Figs. 4 and S3). Consequently, with the same temperature conditions, we compared the production profiles of Strain CgIBR-1 and CgIBR-2 with increased incubation time to analyze the effect of incubation time on the production of APG. Production rates were monitored until 48 h. Interestingly, the increase in incubation time had no significant effect on the production of APG2, APG3, and APG4 with strain CgIBR-1 which maintained a higher level of APG1 for the process of incubation. Conversely, the increased incubation time with strain CgIBR-2 significantly enabled a higher conversion of AP2, APG3 and APG4 whilst reducing the concentration of APG1 (Fig. 5A and 5B)

### Elucidation of Structures of Apigenin Glucosides

**Fig. 6:**
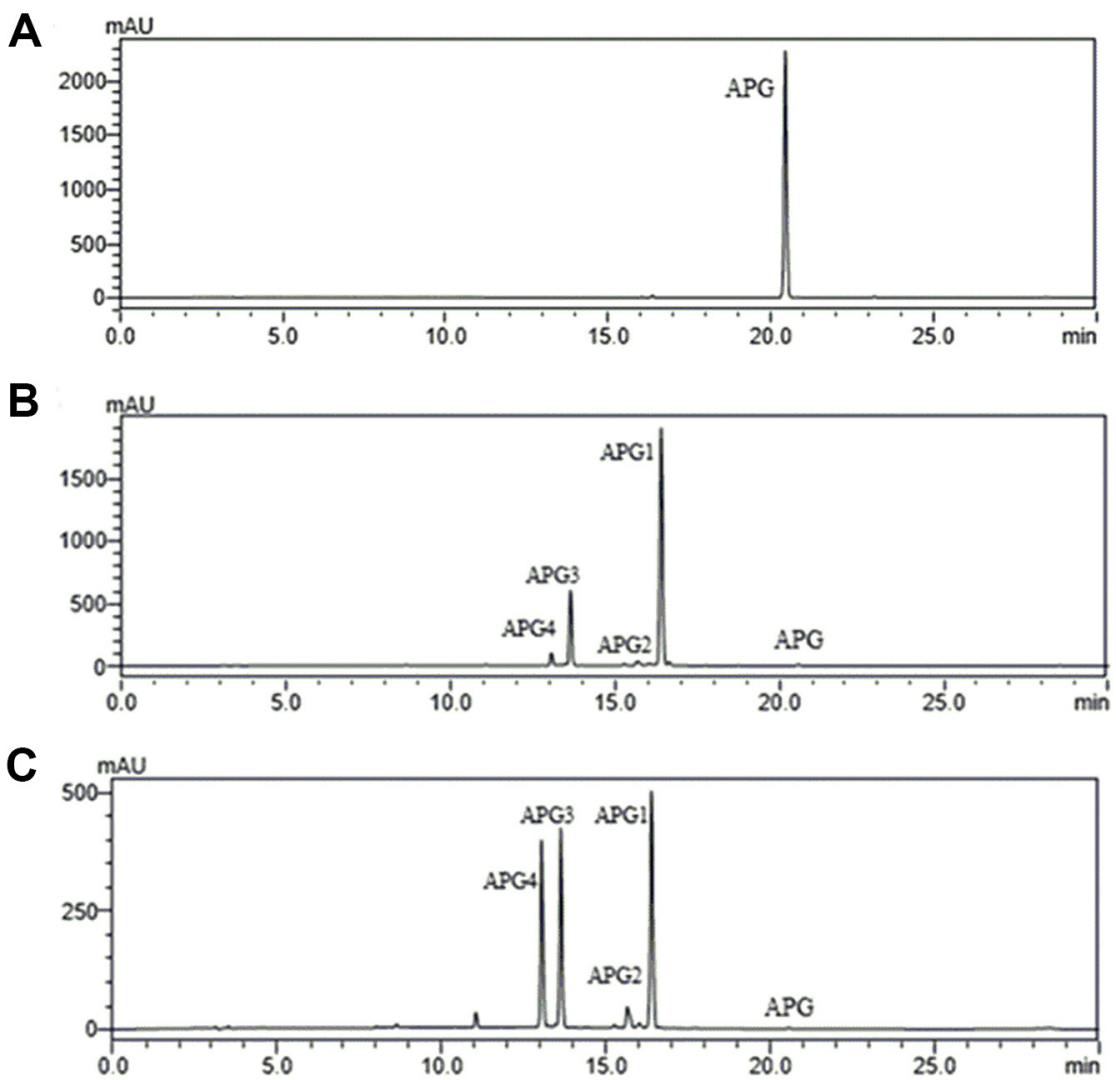
Whole-cell bioconversion of apigenin (APG) to its glucosides (APG1, APG2 APG3, and APG4) in using *C. glutamicum* strains CgIBR-1 and CgIBR-2 after 25 h with 2 mM apigenin. HPLC-PDA chromatogram analyses of (**A**) apigenin standard (APG) and (**B**) biotransformation with CgIBR-1 compared to (**C**) biotransformation with CgIBR2.

**Fig. 7:**
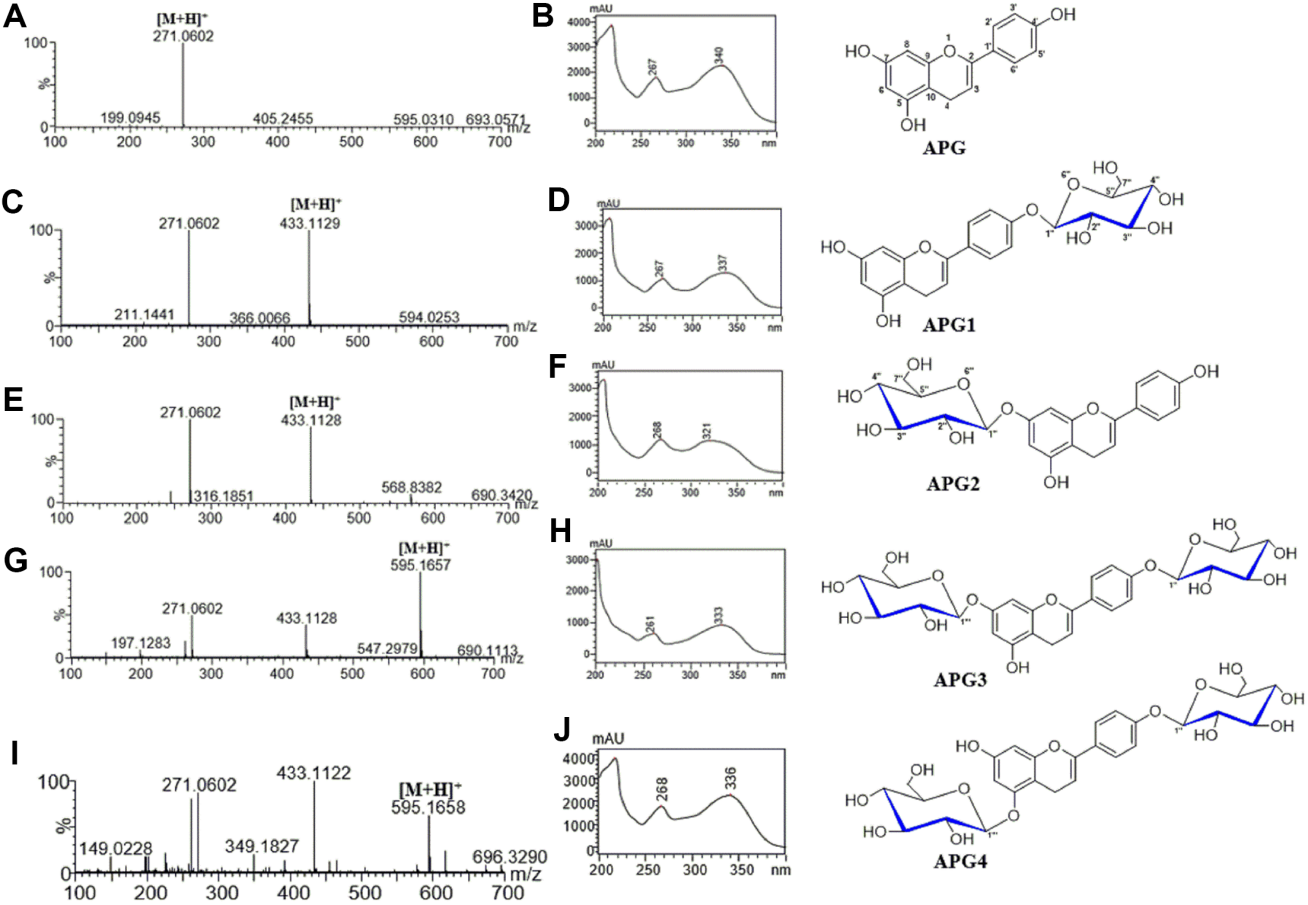
HR-QTOF ESI/MS chromatogram of (A) apigenin standard with (B) UV/VIS of apigenin standard and monoglucosides (C) HR-QTOF ESI/MS chromatogram of monoglucoside APG1 and its (D) UV/VIS spectra; (E) HR-QTOF ESI/MS chromatogram of monoglucoside APG2 and its (F) UV/VIS spectra; (G) HRQTOF ESI/MS chromatogram of diglucoside APG3 and its (H)UV spectra; (I) HR-QTOF ESI/MS chromatogram of diglucoside APG4 and it’s (J) UV spectra.

HPLC-PDA of the ethanolic extracted samples from both strains was carried out under the conditions mentioned in the materials and methods section with standard apigenin. The observed glucosylated products, APG1, APG2, APG3, and APG4 were revealed at a retention time of 16.4 min, 15.7 min, 13.6 min, and 13.1 min respectively (Fig. 6). Furthermore, during preparation of HPLC analysis for separation and purification, the retention times of APG1, APG2 APG3, and APG4 appeared at 17.5 min, 17.1 min, 16.4 min, and 13.9 min respectively. Additionally, when the apigenin standard and our various purified products were analyzed by LC-QTOF-ESI/MS in positive ion mode, the mass spectra displayed an exact mass (*m/z*^+^) of [M+H]^+^ of 271.060 for apigenin. Further analysis of our purified products confirms APG1 and APG2 to be mono glucosides with molecular ion [M+H]^+^ peaks at *m/z* of 433.1129 and 433.1130 respectively. APG3 and APG4 were analyzed and confirmed to be diglucosides with a molecular ion [M+H]^+^ peak at *m/z* of 595.16 (Fig. 7).

Furthermore, to further determine the chemical structure of APG1, APG2, APG3, and APG4, the purified products were subjected to nuclear magnetic resonance. When the ^1^H NMR of APG1 was analyzed, it revealed the presence of signals at δ_H_ 12.94 (1H, s, 7-OH) and δ_H_ 10.45 (1H, s, 5-OH) confirmed the glucosylation at C-4'position. The anomeric proton signal at δ_H_ 5.12 (1H, d, *J* = 7.6 Hz) was consistent with the presence of a β-glucoside unit in APG1. Similarly, the anomeric carbon peak of ^13^C-NMR appeared at 100.3 ppm. Therefore, APG1 was determined to be apigenin-4'-O-β-glucoside. Additionally, the ^1^H NMR spectrum analysis of APG2 showed the presence of signals at δ_H_ 10.50 (1H, s, 4'-OH) and δ_H_ 12.95 (1H, s, 5-OH) also confirmed the attachment of glucose to the C-7 position. The anomeric proton signal at δ_H_ 5.12 (1H, d, *J* = 7.6 Hz) and the anomeric carbon peak, ^13^C-NMR appearing at 104.2 ppm with analyzed QTOF-ESI/MS value of APG2 revealed the chemical structure to be apigenin-7-O-β-glucoside (Fig. 7e). In the same way, in the ^1^H-NMR analysis of APG3, two proton peaks showed chemical shift values of δ 12.95 ppm (7-OH) and δ 10.6 ppm (4'-OH), indicating the attachment of glucose to the 7-OH and 4'-OH positions. In the ^13^C-NMR analysis, anomeric carbon peaks appeared at 100.3 ppm and 100.1 ppm. The anomeric proton signal at δ_H_ 5.05 (d, *J* =7.6 Hz) and δ_H_ 5.08 (d, *J* = 7.6 Hz), revealed the structure of APG3 to be apigenin-4',7-O-β-diglucoside. For APG4, the 1H-NMR revealed the presence of a signal at H 10.89 (1H, s, 7-OH) and two proton peaks showed chemical shift values of δ 10.5 ppm (5-OH) and δ 10.6 ppm (4'-OH). The anomeric proton signals δ_H_ 5.13 (d, *J* =7.4 Hz) and 4.8 (d, *J* =7.6 Hz) and the anomeric carbon peaks ^13^C-NMR of 104.1 ppm and 103.6 ppm revealed APG4 to be a non-natural apigenin-4',5-O-β-diglucoside. Details of ^1^H and ^13^C data and spectra are shown in Tables 1 and 2 and Figs. S4-S13.

## Discussion

Apigenin has three phenolic hydroxyl groups at the C-5, C-7 , and C-4' positions, and previous reports have confirmed its stability in the form of mono-, di-, and tri-glucosides in different species of plants [43]. The synthesis of glucosides has usually been carried out by chemical approach which presents numerous disadvantages such as the employment of different reaction conditions for the synthesis of various derivatives, strict monitoring of reaction procedures, protection of unreacted moiety, and difficulty in purification [44-46]. Recently, apigenin glucosides were enzymatically synthesized by Rit *et al*. [47], however, enzymatic synthesis, involves the use of expensive NDP-sugars which makes it quite impossible to large-scale synthesis for the demand of human use. The engineering of microbial cells and their subsequent application as cell factories for the production of a wide range of valuable products serves as a formidable means for the eco-friendly production of medicinal compounds by simple fermentation [48]. Additionally, by using the biosynthetic approach with GTs to gluco-diversify flavonoids, natural as well as non-natural compounds have been developed which could have novel properties [49]. The overexpression of promiscuous enzymes such as YdhE coupled with the usage of endogenous UDP-sugar donors in *C. glutamicum* could reduce the expense of engineering whole-cell *E. coli* systems for the in vivo biosynthesis of flavonoid glucosides.

For years, *C. glutamicum* has proven to be a versatile platform for the production of amino acids, particularly glutamate, lysine, and other biofuels [26]. The ability to produce high titers of flavonoid derivatives adds to the ongoing research on the extent to which the *C. glutamicum* cell factory system is versatile. *C. glutamicum* was able to withstand substrate toxicity as it was able to glucosylate apigenin in higher concentrations of up to 12 mM which is almost ten times that of engineered *E. coli* (Fig. 3). Additionally, based on the expression of GTs in *E. coli*, the titers of flavonoid glucosides were less than 100 mg/l, probably due to the low production of intracellular UDP-glucose [50]. The simple overexpression of UDP-glucose pathway genes *galU1* and *pgm* greatly improved the titers of all products.

Furthermore, the capability of *C. glutamicum* to grow in a wide range of temperatures allows for a simple control mechanism of production of various products and glucosides, without any additional metabolic stress. Similarly, the fermentation products of glutamate and other products were regulated and improved when temperatures were regulated in *C. glutamicum* [51, 52]. In this case, Low temperatures favor the production of monoglucosides while diglucosides are favored by comparatively higher temperatures. Here we have experimentally biosynthesized apigenin derivatives APG1, APG2, APG3, and a novel non-natural APG4 from apigenin using *C. glutamicum* cell factory system expressing a promiscuous glycosyltransferase, YdhE from *Bacillus licheniformis*. The maximal yield of each metabolite was 4.2 mM, 0.6 mM, 1.7 mM, and 2.1 mM respectively. A literature review on apigenin and its glucosides, APG1, APG2, and APG3 reveals their anti-cancer, anti-proliferative, anti-inflammatory, and anti-oxidant properties [53-55]. Similarly, the newly synthesized APG4 could possess enhanced activity compared to the already-known derivatives.

## Conclusion

In this study, we report the use of *C. glutamicum* cell factory as a versatile chassis for the in vivo production of apigenin glucosides from glucose using simple metabolic engineering tools. The introduction of heterologous GT and upregulating the biosynthetic pathway of UDP-glucose enabled a higher efficiency for the production of apigenin glucosides. The system ensures a high titer yield with the control of production towards different glucosides, making it feasible and easy for industrial-scale biosynthesis of APG glucosides. Additionally, the status of *C. glutamicum* as GRAS makes it advantageous for the production of apigenin and other flavonoid glucosides for human consumption. The high production of endogenous UDP-glucose, high substrate susceptibility and the ability to control production towards preferred glucosides render the system a promising platform for sustainable, eco-friendly, non-toxic, and industrial-scale production of apigenin and other flavonoid derivatives.

## Supplemental Materials

Supplementary data for this paper are available on-line only at http://jmb.or.kr.



## Figures and Tables

**Table 1:** ^1^H-NMR analysis of apigenin and its glucoside derivatives.

Proton/δppm	Apigenin	APG1	APG2	APG3	APG4
1	-	-	-	-	-
2	-	-	-	-	-
3	6.76	7.25	7.20	7.18	7.08
4	-	-	-	-	-
5	-	-	--	-	-
6	-	-	-	-	-
7	6.48	6.83	6.74	6.63	6.71
8	-	-	-	-	-
9	6.19	6.44	6.64	7.03	6.93
10	-	-	-	-	-
1'	-	-	-	-	-
2'	7.91	7.94	7.93	7.97	8.13
3'	6.93	6.94	7.25	7.24	7.23
4'	-	-	-	-	-
5'	6.93	6.94	7.25	7.24	7.23
6'	7.91	7.94	7.93	7.97	8.13
5-OH (12)	12.95	12.94	12.95	12.85	-
7-OH (11)	10.51	10.45	-	-	10.85
4'-OH (14)	10.68	-	10.50	-	-
1''	-	5.34(d, *J*=7.6Hz)	5.18(d, *J*=7.1 Hz)	5.05(d, *J*=7.6Hz)	5.12(d, *J*=7.4Hz)
1'''	-	-	-	5.08(d, *J*=7.6Hz)	4.82(d, *J*=7.6Hz)
Sugar protons	-	3.18-5.18	2.50-4.78	2.85-4.26	2.52-4.25

**Table 2:** ^13^C-NMR analysis of apigenin and its glucoside derivatives.

Carbon/δppm	Apigenin	APG1	APG2	APG3	APG4
1	-	-	-	-	-
2	164.24	163.39	169.37	165.58	165.29
3	103.28	103.54	104.67	104.56	108.20
4	182.21	182.46	175.80	182.25	177.28
5	104.14	105.79	105.10	105.93	110.11
6	161.61	161.82	166.68	164.15	161.47
7	99.31	99.99	99.71	100.12	99.93
8	164.61	164.75	165.76	165.58	161.87
9	94.46	95.32	98.37	97.35	99.33
10	157.78	157.40	158.92	161.56	158.55
1'	121.64	121.47	126.50	124.28	128.56
2'	128.93	129.09	128.43	128.81	131.15
3'	116.44	116.48	116.42	117.13	115.46
4'	161.89	161.55	165.76	163.54	161.62
5'	116.44	116.48	116.42	117.13	115.46
6'	128.93	129.09	128.43	128.81	131.15
1''	-	100.36	104.22	100.02	104.07
				99.99	103.64
Sugar carbon	-	62.07-77.60	59.32-77.62	61.14-77.61	61.60-77.84
