# Supplementary material for: Biosynthesis of Apigenin Glucosides in Engineered Corynebacterium glutamicum
Source: J Microbiol Biotechnol. 2024 Mar 14;34(5):1154–63. doi: 10.4014/jmb.2401.01017 (PMC11180926; doi:10.4014/jmb.2401.01017)
Supplement: Supplementary file 1 [file jmb-34-5-1154-supple.pdf]

Supplementary Information

Biosynthesis of apigenin glucosides in engineered *Corynebacterium glutamicum*

Obed Jackson Amoah[1], Samir Bahadur Thapa[1], Su Yeong Ma[1], Hue Thi Nguyen[1], Morshed Md Zakaria[1], Jae Kyung Sohng[1,2]**

[1]*Department of Life Science and Biochemical Engineering, Sun Moon University, 70 Sunmoon-ro 221, Tangjeong-myeon, Asan-si, Chungnam 31460, Republic of Korea*

[2]*Department of Pharmaceutical Engineering and Biotechnology, Sun Moon University, 70 Sunmoon-ro 221, Tangjeong-myeon, Asan-si, Chungnam 31460, Republic of Korea*

Corresponding author:

Prof. Jae Kyung Sohng

Tel: +82(41)530-2246

Email: sohng@sunmoon.ac.kr

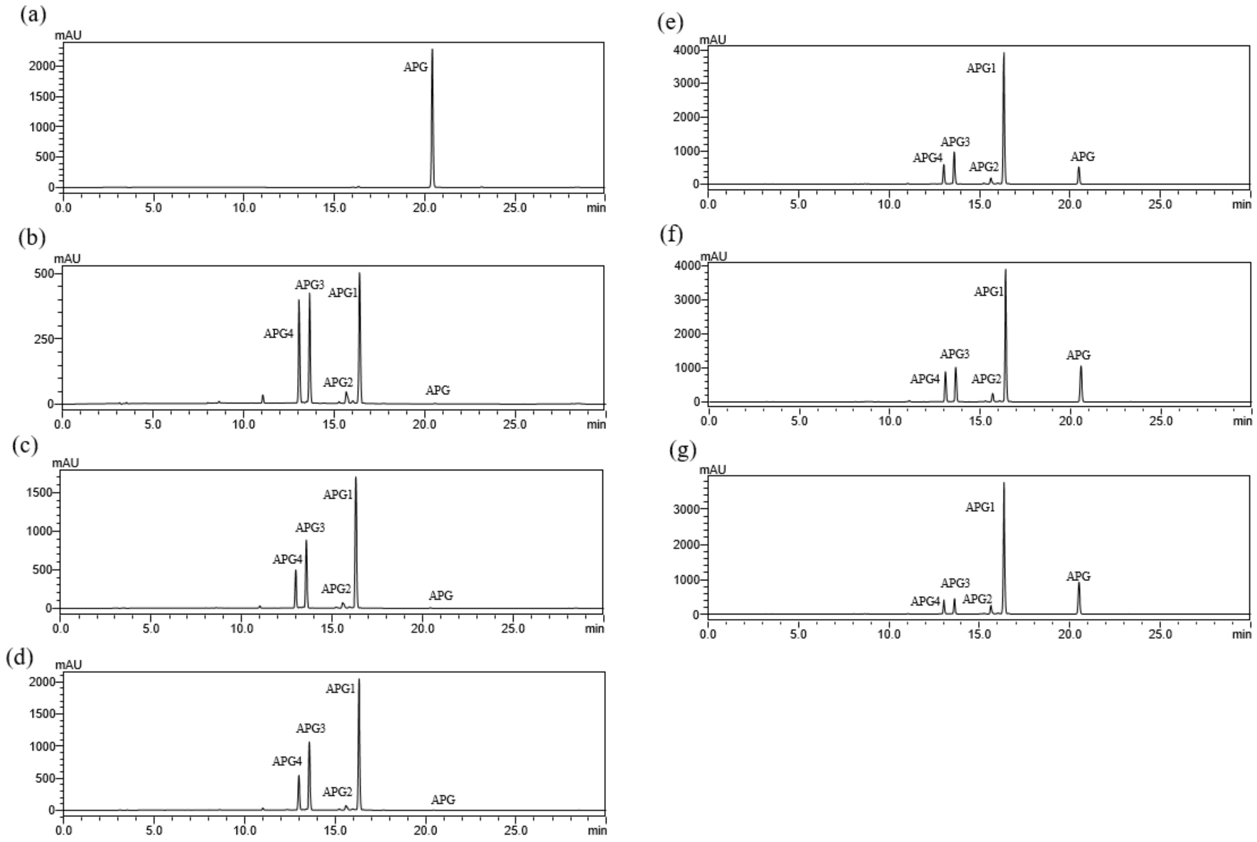

Fig. S1. HPLC profiles for the production of apigenin glucosides with different concentrations of apigenin; (**a**) Apigenin standard; (**b**) 2 mM; (**c**) 4 mM; (**d**) 5 mM; (**e**) 8 mM; (**f**) 10 mM and (**g**) 12 mM with strain CgIBR-2

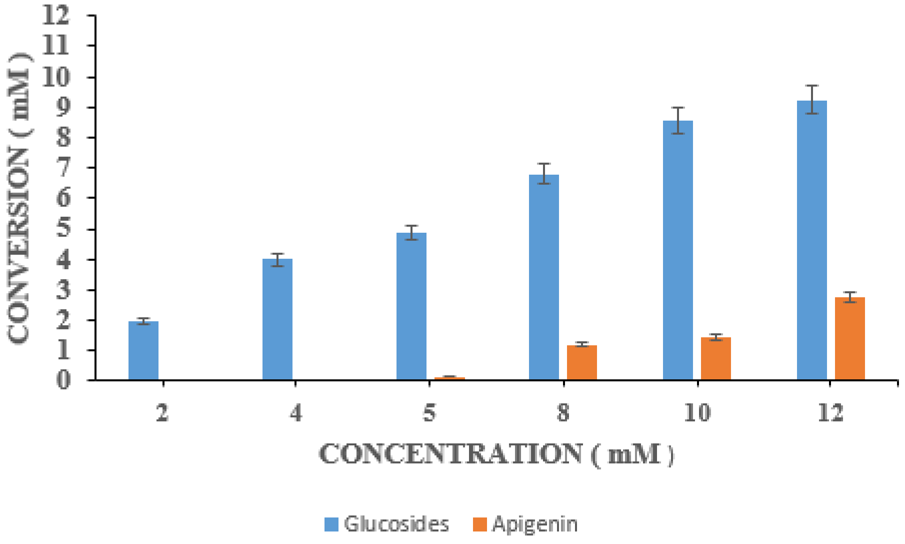

Fig.S2. Conversion profiles of Apigenin glucosides compared with residual apigenin concentrations in the culture medium

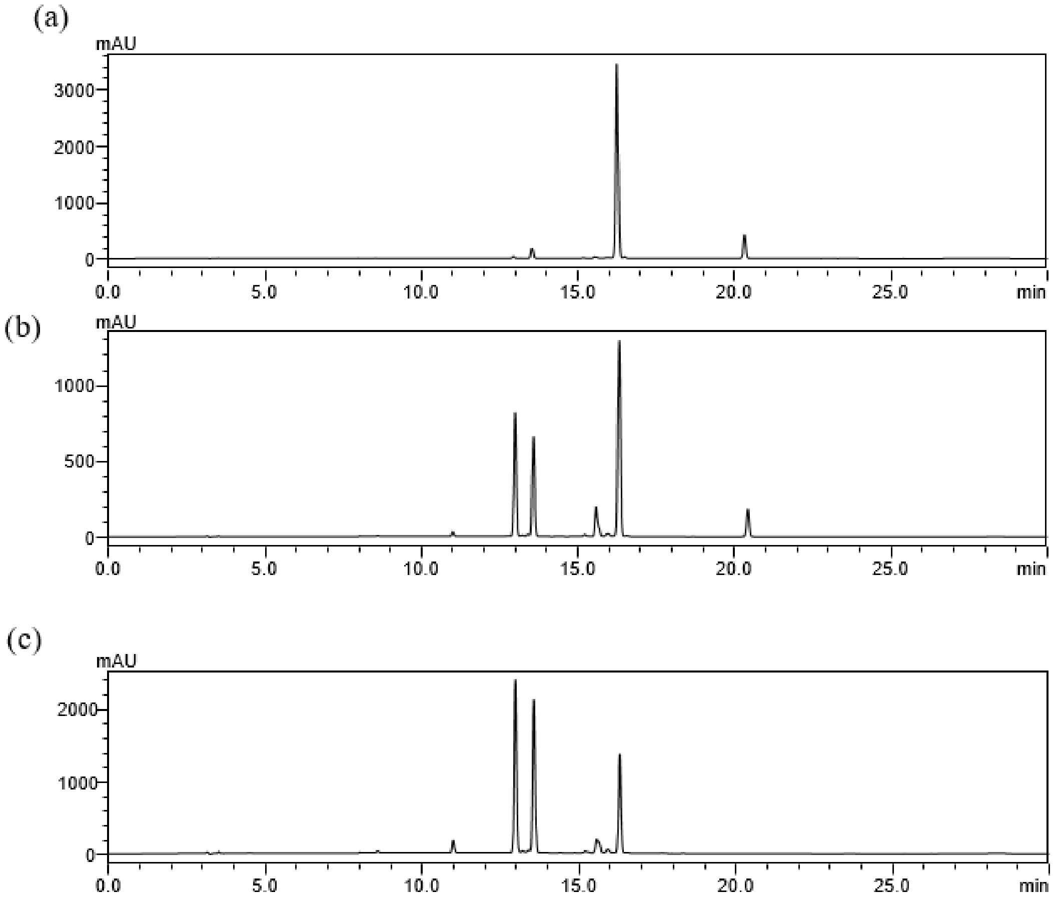

Fig. S3. HPLC profile for the production of apigenin glucosides under different temperatures conditions with 5 mM of apigenin; (**a**) 25°C; (**b**) 33°C and (**c**) 37°C.

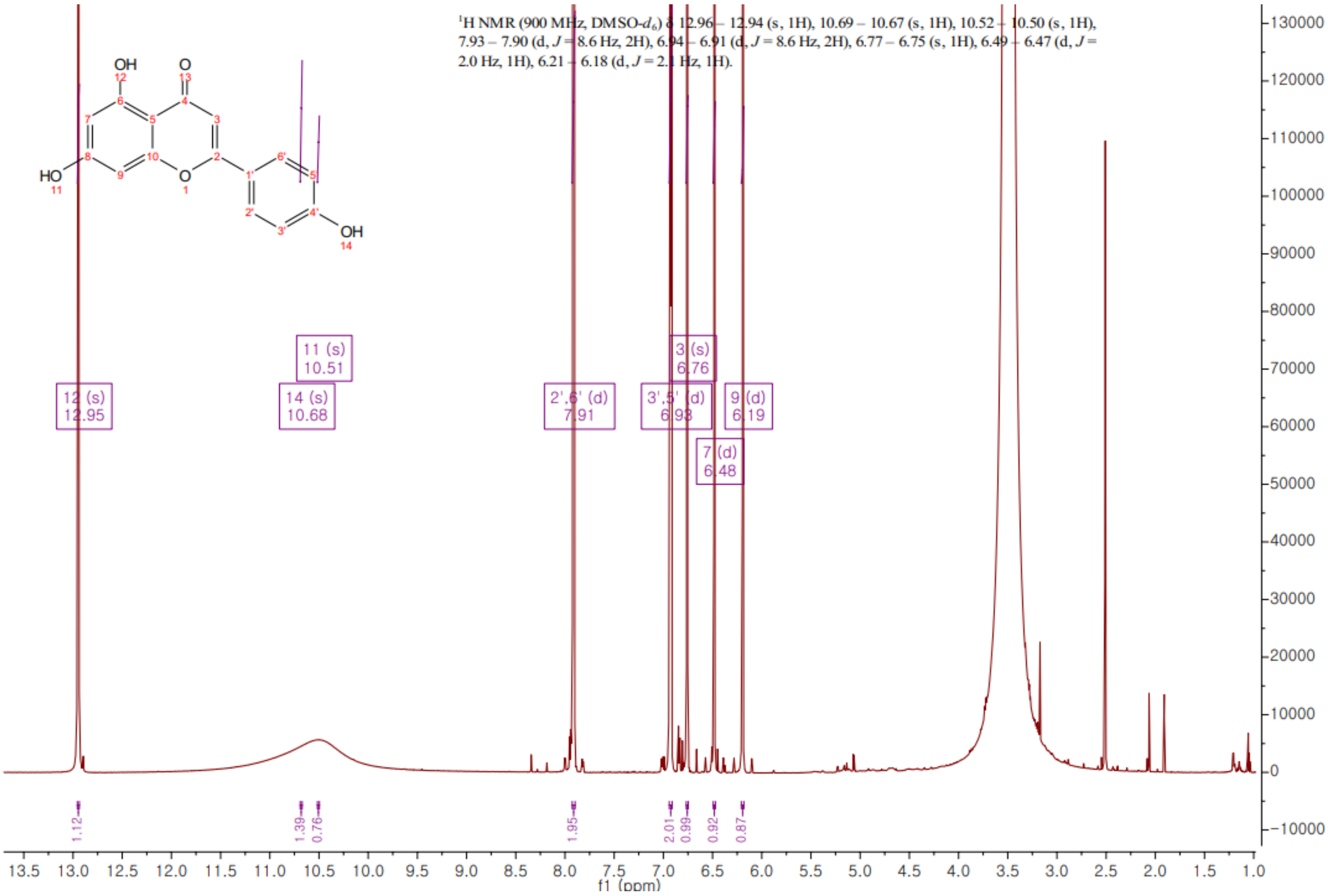

Fig. S4; ^{1}H NMR spectra of apigenin standard

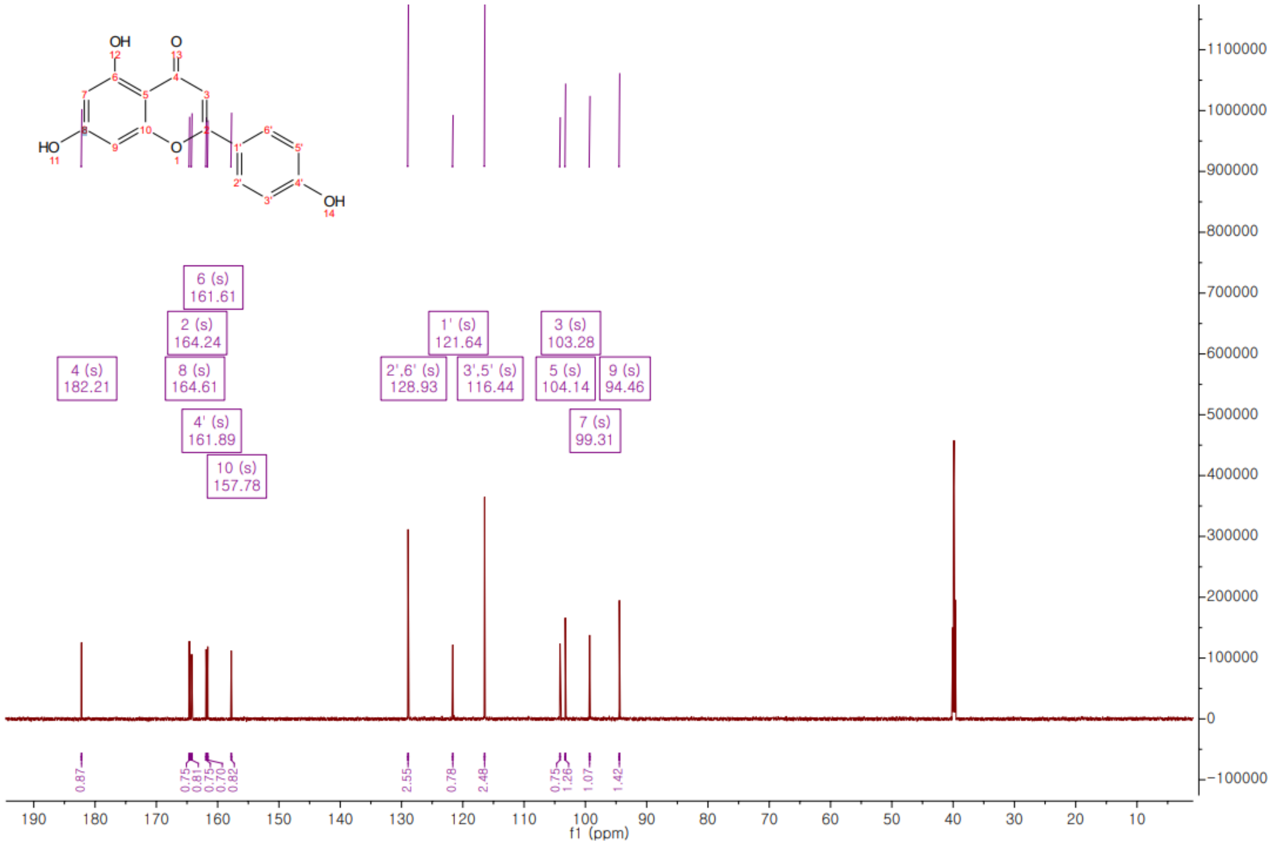

Fig. S5; ^{13}C NMR of apigenin standard

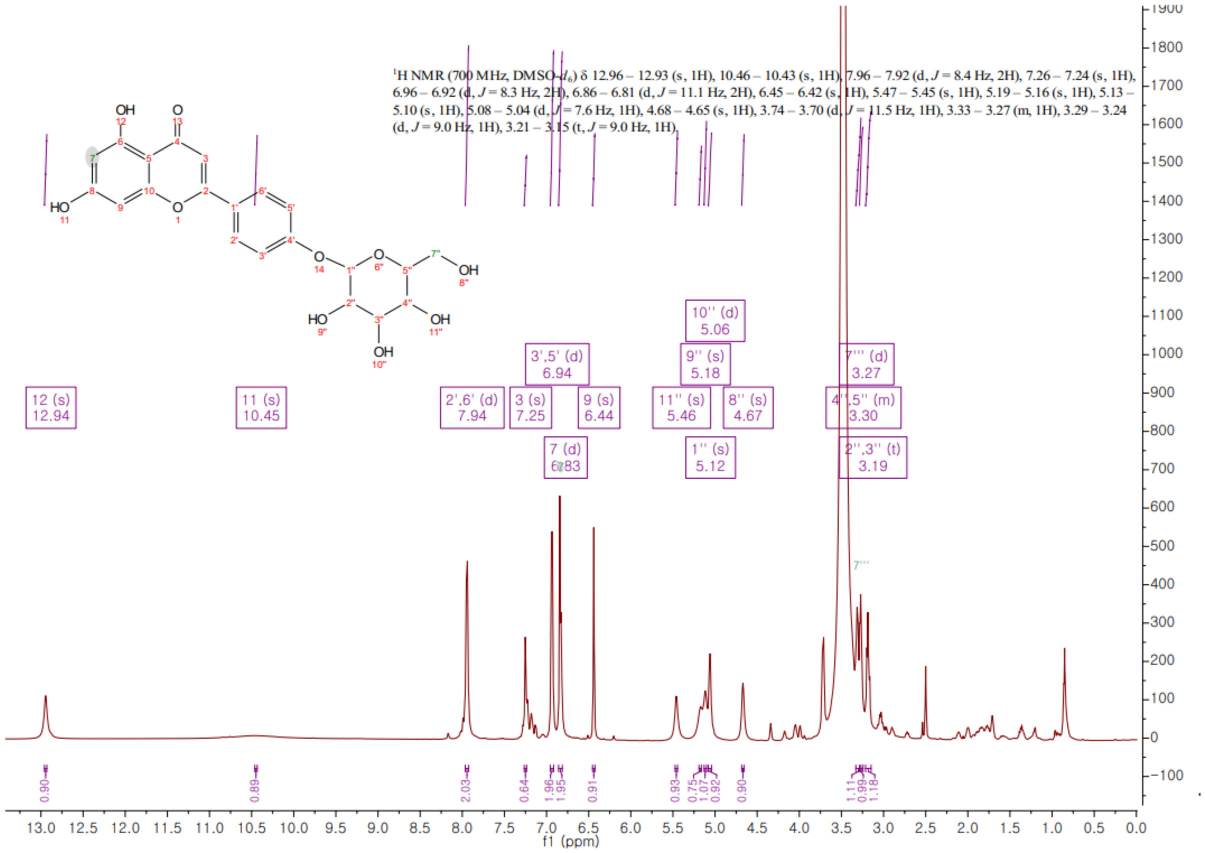

Fig. S6; ^{1}H NMR spectra of APG1

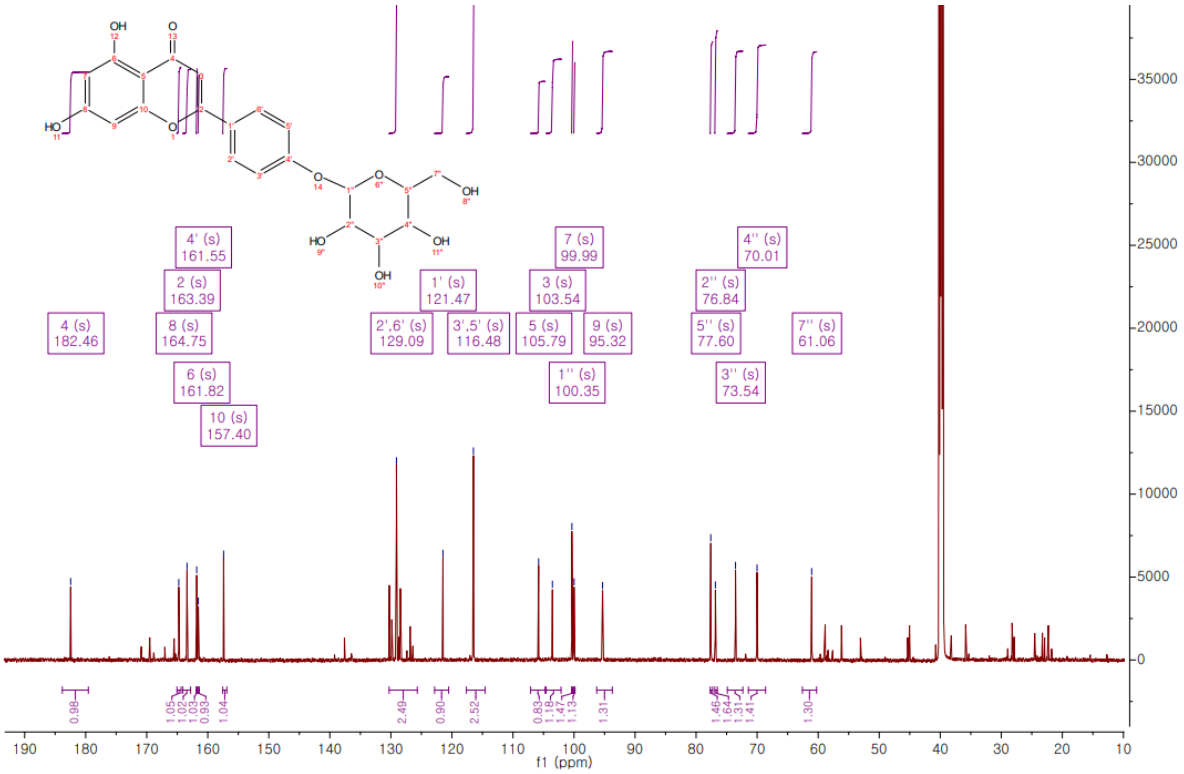

Fig. S7; ^{13}C NMR Spectra of APG1

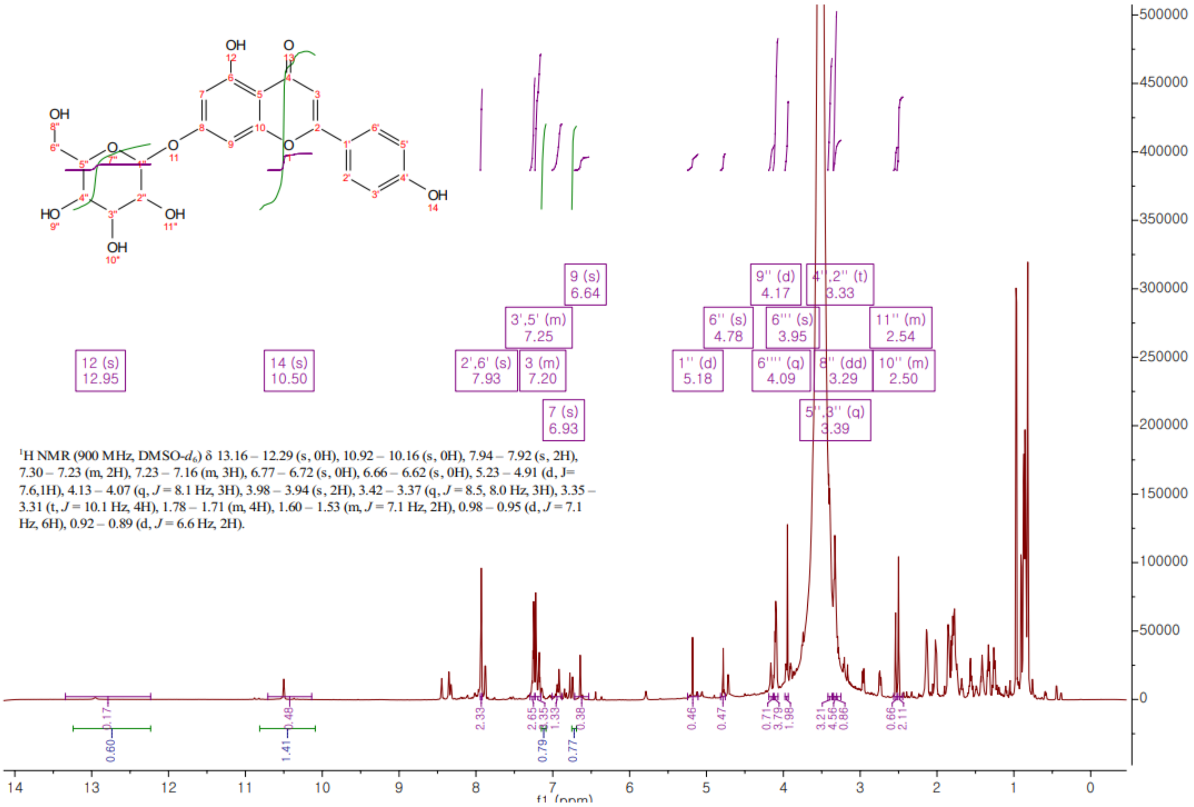

Fig. S8; ^{1}H NMR spectra of APG2

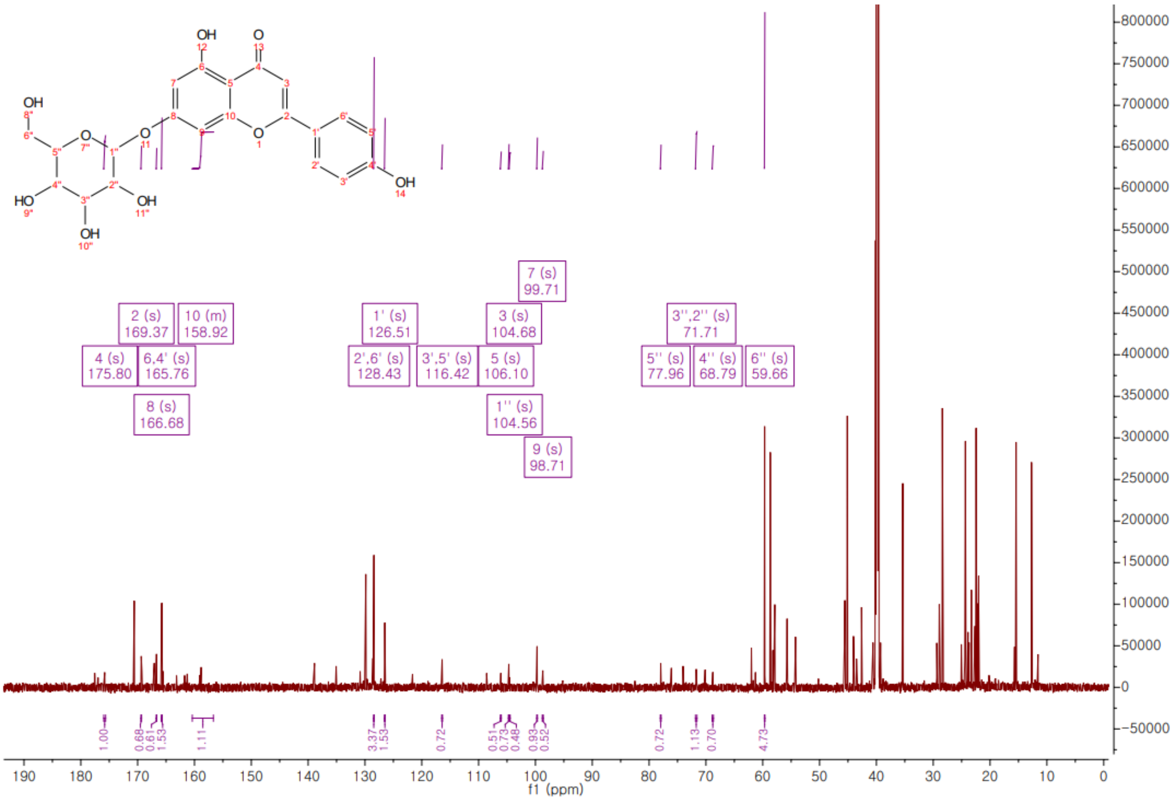

Fig. S9; ^{13}C NMR Spectra of APG2

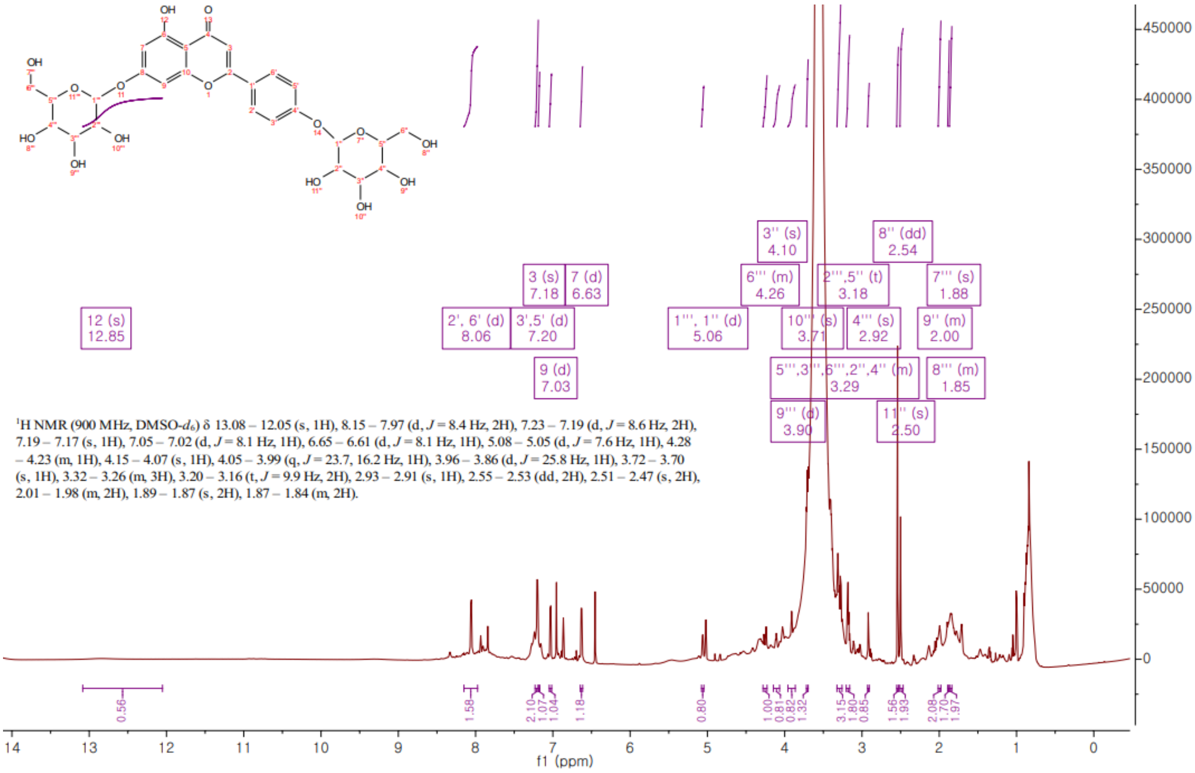

Fig. S10. ^{1}H NMR spectra of APG3

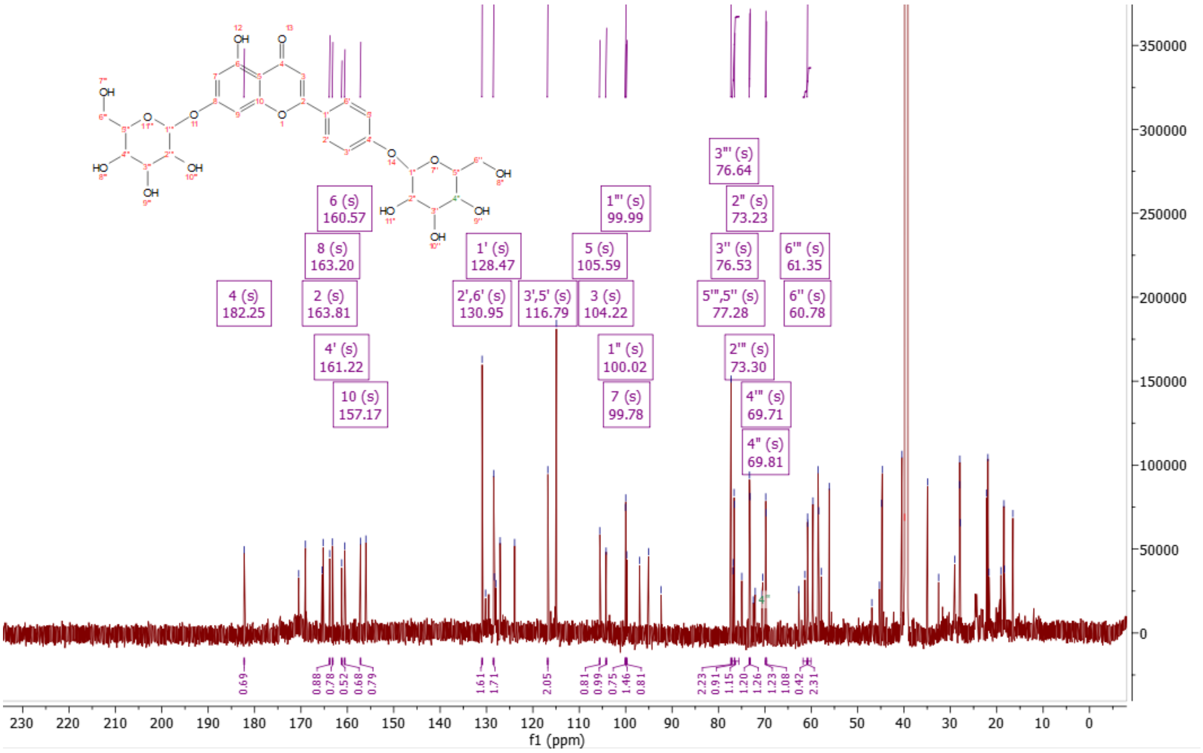

Fig. S11. ^{13}C NMR Spectra of APG3

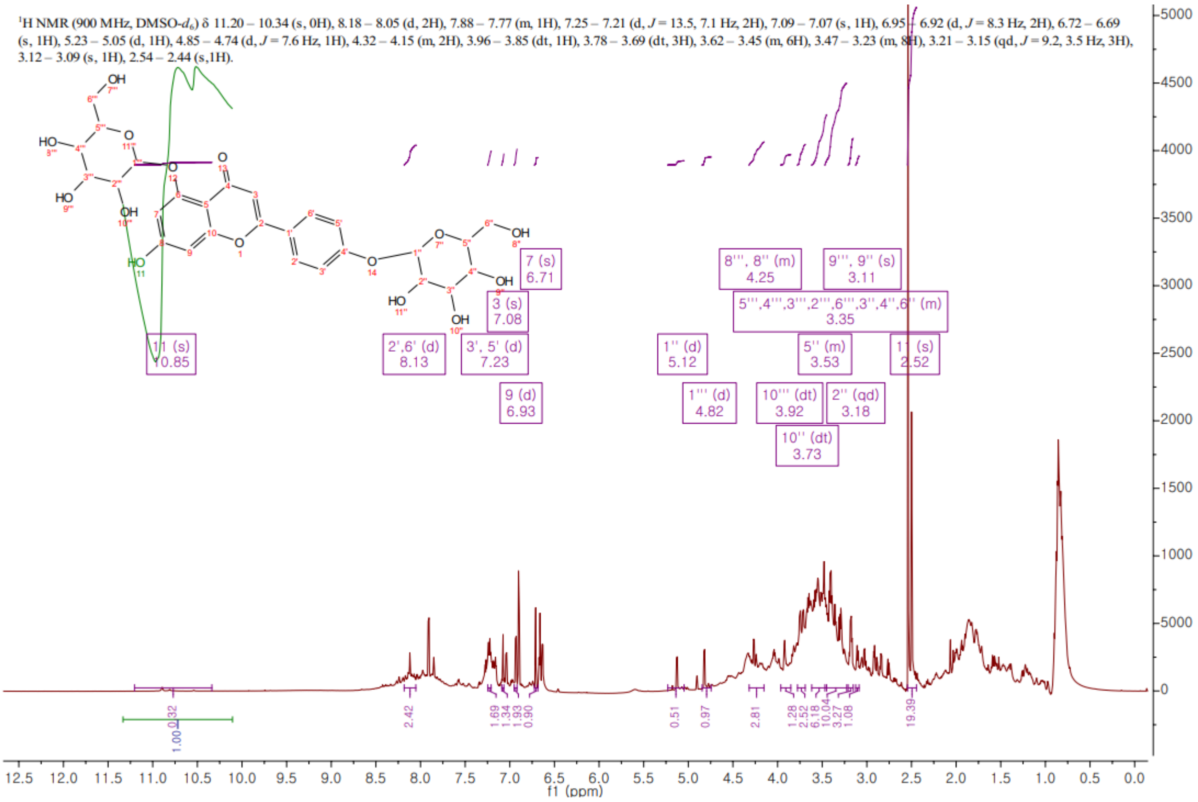

Fig. S12. ^{1}H NMR spectra of APG4

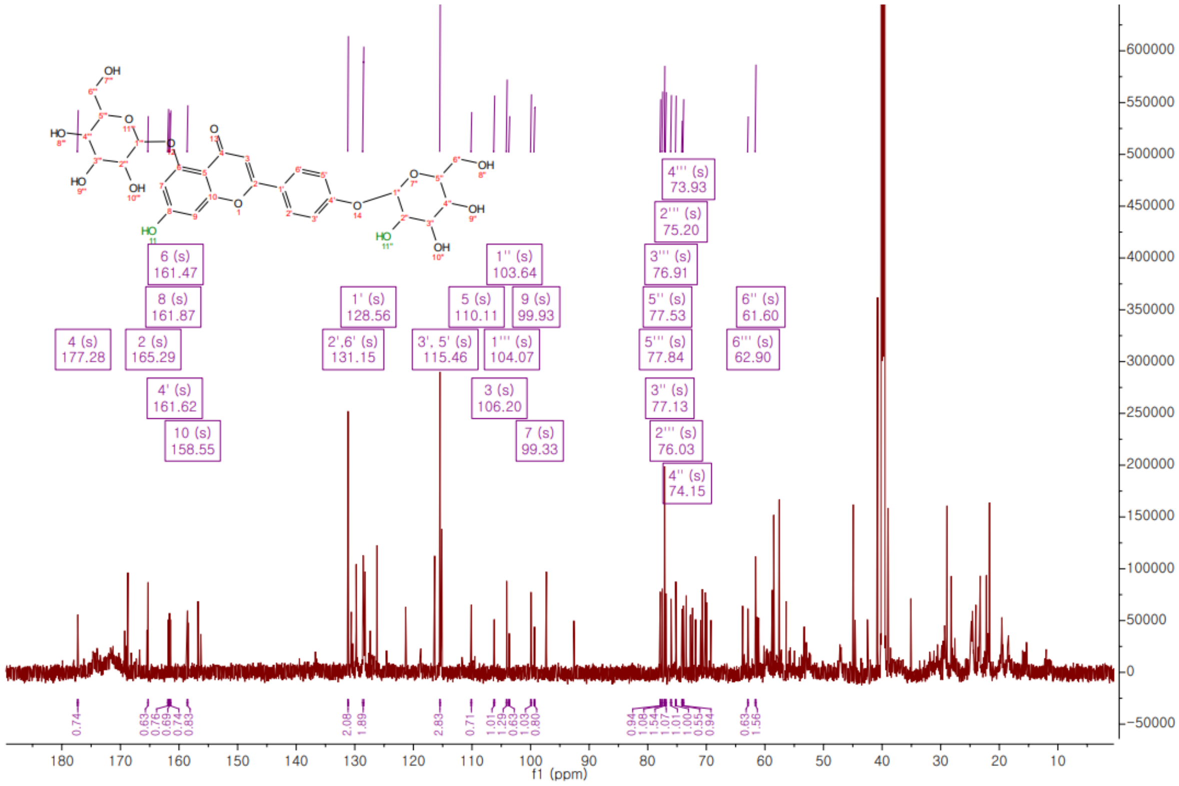

Fig. S13. ^{13}C NMR Spectra of APG4

Table S1; Strains and plasmids used

Strains and plasmids	Relevant Characteristics	Source or references
**Strains**		
E. coli XL1Blue	*Δ(mcrA)183 Δ(mcrCB-hsdSMR-mrr)173 endA1 supE44 thi-1 recA1 gyrA1 gyrA96 relA1 lac* [F' proAB *lacIqZΔM15* Tn10 9Tetr)]	Stratagene
C. glutamicum	Wild type strain, ATCC 13032	ATCC
CgIBR- 1	*C. glutamicum* harboring pSKSM-Y	This study
CgIBR- 2	*C. glutamicum* harboring pSKSM-YGP	This study
**Plasmids**		
pGEM®-T easy vector	*E. coli* general cloning vector, amp^r	Promega (USA)
pSK003	Km^R; *C. glutamicum/E. coli* shuttle vector. (P_{sod}, pBL1, $oriV_{C.g.}$, $oriV_{E.c}$).	Our lab
pSKSM	pSK003+P_{tac}+LacI	[39]
pSKSM-Y	pSK003+P_{tac}+LacI+*ydhE*	This study
pSKSM-YGP	pSKSM harboring *ydhE*, *galU1*, and *pgm*	This study

Table S2; Primers used for this study

Name	Sequence (5'-3')
GalU1-F	TAGTCTAGAAAGGAGATATACAATGAGTTTGCCTATCGATGAG
GalU1-R	CTGCGGCCGCCTATTTTACTTGAGAATCGTCTGCAATACG
Pgm-F	CTGCGGCCGCAAGGAGATATACAATGGCACATGAACGCGCCGG
Pgm-R	AGCTGCAGTTACTGTCCGAGTACTTCGCTGACCAA
